# Response of soil microbial community to plant composition changes in broad-leaved forests of the karst area in Mid-Subtropical China

**DOI:** 10.7717/peerj.12739

**Published:** 2022-03-07

**Authors:** Liling Liu, Ninghua Zhu, Guangyi Zhou, Peng Dang, Xiaowei Yang, Liqiong Qiu, Muyi Huang, Yingyun Gong, Suya Zhao, Jie Chen

**Affiliations:** 1 Central South University of Forestry and Technology, Changsha, China; 2 Research Institute of Tropical Forestry, Chinese Academy of Forestry, Guangzhou, China; 3 Jianfengling National Key Field Research Station for Tropical Forest Ecosystem, Hainan Island, China

**Keywords:** Vegetation composition, Microbial community, Soil nutrients, Plant diversity, Karst ecosystem, *Cryptomeria japonica*

## Abstract

The rapid growth and expansion of*Cryptomeria japonica* (Thunb. ex L. f.) D. Don in karst area strongly affects plant composition of native deciduous broad-leaved forest, which seriously threat ecosystem function and service. Given the importance of soil microorganisms in regulating nutrients cycling and plant species coexistence, understanding soil microbial attributes and their relationships with soil and vegetation features in forests harboring different *C. japonica* abundance will help understanding the drivers of ecosystem function changes. Here we examined the diversity and composition of soil bacterial and fungal communities and their correlations with plant diversity as well as soil physicochemical properties in karst broad-leaved forests with different relative abundances of *C. japonica* (i.e., a high, moderate, low and no proportion level with a stem density of 1,487, 538, 156 and 0 plant/hm^2^, respectively) in Mid-Subtropical China. We found that soil pH decreased while soil water content (SWC), total nitrogen (TN), total phosphorus (TP) and total potassium (TK) tended to increase with the increase in *C. japonica* abundance. In contrast, soil available nitrogen (AN), available phosphorus (AP) and available potassium (AK) content declined by 26.1%∼49.3% under the high level of *C. japonica* abundance. A gradual decrease in relative abundance of Acidobacteria and Chloroflexi while a pronounced increase in relative abundance of Ascomycota and Basidiomycota were observed with increase of *C. japonica* abundance. Alternations in bacterial composition were closely related to changes in AP and AK, while the change of fungal structure was mainly related to SWC, soil organic carbon (SOC) and pH, indicating that bacterial community was sensitive to declines in soil available nutrients and fungal structure was sensitive to changes in soil physicochemical properties (i.e., pH and SWC) and organic carbon resource. Understory plants had the highest *α*-diversity in forest containing moderate abundance of *C. japonica*, which might be related to the high bacterial diversity. Our findings suggest conservation of soil bacterial and fungal taxa that are responsible for nutrients availability and carbon sequestration is of great significance for improving the resistance of natural deciduous broad-leaved forests to the rapid spread of *C. japonica* in karst areas. Moreover, Acidobacteria, Chloroflexi, Ascomycota and Basidiomycota are potential indicators for soil properties changes, which should be taken into consideration in karst forest managements.

## Introduction

Soil microorganisms play a leading role in element cycling and energy flow through trophic levels, maintaining the stability and sustainability of forest ecosystem (Cao & Ni, 2015). In particular, soil microorganisms drive the mineralization process of soil organic matter (Shao et al., 2021), and play an important role in nutrients immobilization and C sequestration (Dai, Yan & Xie, 2017). Soil microbial biomass is an important part of soil organic C and N pools (Paul, 2015). The activity, species diversity, functional and taxonomic composition of soil microorganisms are sensitive to climate, ecosystem types, soil physical and chemical properties and plant species composition (Bainard et al., 2014; Hazard et al., 2013), which in turn affects the soil health and quality. Therefore, microbial community attributes are potential indicators of soil nutrient pool size and nutrient availability (Deng, Cheng & Hui, 2016; Riah, Trinsoutrot & Martin, 2015). Understanding the alternation of microbial attributes, especially the diversity and functional characteristics, in response to environmental changes in any complex ecosystem can help to improve the ability of predicting ecosystem responses to external changes (Niu et al., 2017).

Soil is the medium of plant interaction, and plants have a significant impact on land productivity mainly by changing soil properties (Pyšek et al., 2020). Plants can affect soil microbial diversity and composition through direct competition for nutrient resources and indirect influence on soil physiochemical environment (Vilà et al., 2011). The vegetation composition will affect the symbiotic relationship between vegetation and soil microorganisms, change the structure and function of microbial community, and alter soil properties. Peng et al. (2019) summarized that the existence of *Phyllostachys edulis* in broad-leaved forest can increase soil microbial biomass and diversity, which may be attributed to changes in soil organic carbon pool. It is also possible that after the emergence of *Phyllostachys edulis*, the activity of soil microorganisms capable of degrading allelochemicals decreased, afterwards the degradation rate of allelochemicals slowdown, which can affect the growth of other plants (Li et al., 2017). In addition, soil microorganisms can have a cascade effect on herbivorous insects and indirectly regulate vegetation composition through the food chain (Lu, He & Ding, 2018). In view of the pivotal role of soil microorganisms in regulating soil nutrient availability and maintaining ecosystem stability, microbial responses to changes in vegetation composition are important in predicting the effects of different vegetation composition on forest restoration. Although effects of plant composition changes on soil microbial community have been widely discussed in previous studies, there are still controversies on these effect modes, and the importance of different impact modes varies greatly under different plant species.

*Cryptomeria japonica* that harbors fragrant wood, water-proof and corrosion-resistant qualities, has the features of wind prevention, sand fixation and water and soil conservation, and the expansion ability is strong in the surrounding stands. The growth space of deciduous broad-leaved forest in karst area have been seriously threatened by the growth and expansion of *C. japonica*. Understanding the effects of different plant composition on both above and below ground communities, in particular the soil microbial community, is essential for maintaining karst ecosystem functions. However, few studies have examined the effects of different relative abundance of *C. japonica* on plant diversity, soil nutrients content, soil microorganisms and their relationships. The responses of soil microbe-plant continuum to changes in *C. japonica* abundance in deciduous broad-leaved forest in karst area were investigated, aiming to clarify: (1) The structure, diversity and functional composition of soil bacterial and fungal communities under different vegetation composition characterized by different proportion of *C. japonica*; (2) How the changes of soil microbial community relate to alternations in soil properties and plant diversity.

## Materials & Methods

### Study site

The study site is located in the national long-term research base of comprehensive management of rocky desertification in Wuling Mountain, Hunan Province, Mid-Subtropical China (27°44.5′N, 109°10′E). The climate is subtropical monsoon humid climate, with mean annual precipitation of 1,300–1,500 mm, and mean annual temperature of 16.3 °C. Soil is mainly yellow brown developed from limestone, which belongs to moderate karst area. The native vegetation is dominated by broad-leaved forests and main native plant species are *Houttuynia cordata*, *Cryptotaenia japonica* and *Achyranthes bidentate*. After 15 years of changes in vegetation composition, significant changes have taken place in understory plants. According to the stem density of *C. japonica* in broad-leaved forest, the proportion of *C. japonica* can be divided into high (H), moderate (M), low (L) and no (N). Forests stands harboring different *C. japonica* proportion were selected to represent different vegetation composition. The overall stand profile is shown in Table S1.

Three 20 m × 20 m plots were randomly established in different vegetation composition area. The space between any two plots within one type of area was more than 20 m. Plant community and species surveys were conducted in May 2020, and individuals with diameter at breast height (DBH) ≥ one cm were recorded with species name, height, coverage and frequency (Han et al., 2020). Three 2 m × 2 m plots and three 1 m × 1 m plots were arranged in each 20 m × 20 m plot for shrub and herbage investigations, respectively.

### Soil sampling

In August 2020, three 2 m × 2 m quadrats were randomly set in each 20 m × 20 m plot, and three 0–20 cm topsoil samples were randomly collected in each quadrat. The soil samples of each plot were mixed well, and the visible animal and plant residues and small stones were carefully removed. Soil samples were stored 4 °C and immediately taken back to the laboratory for treatment. Soil samples were divided into two parts, one for the analysis of microbial community and the other for the determination of soil physiochemical properties.

Soil organic carbon (SOC) was determined by potassium dichromate sulfuric acid oxidation method. Total nitrogen (TN) was determined by semi-micro Kjeldahl method. Total phosphorus (TP) was determined by molybdenum antimony anti spectrophotometry. Available phosphorus (AP) was determined by sodium bicarbonate extraction colorimetry. Estimation of available nitrogen (AN) mainly adopted magnesium oxide extraction diffusion method. The soil water content (SWC) was measured by oven drying and ring knife method (Bao, 2000).

### Soil DNA extraction

The TGuide S96 magnetic bead method soil genomic DNA extraction kit was used to complete DNA extraction according to the manufacturer’s instructions. A total of 12 DNA samples representing 12 studying plots were obtained in the end. The full length primers for the 16S rRNA region were 27F: (5′-AGAGTTTGATCCTGGCTCAG-3′), and 1492R: (5′-GGTTACCTTGTTACGACTT-3′). The fungal full-length primers used were ITS1F (5′-CTTGGTCATTTAGAGGAAGTAA-3′) and ITS4 (5′-TCCTCCGCTTATTGATATGC-3′) (Edgar, 2013). The DNA sequencing was performed on PacBio platform. The quantitative determination, homogenization, library preparation, Online sequencing and data quality control of PCR amplification products were all completed by BMK Cloud (Biomarker Technologies Co., Ltd., Beijing, China. http://www.biocloud.net) (Joshi et al., in press).

### Bioinformatic analysis

The bioinformatics analysis of this study was performed with the aid of the BMK Cloud (http://www.biocloud.net). The raw reads generated from sequencing were filtered and demultiplexed using the SMRT Link software (v 8.0) with the minPasses ≥5 and minPredicted Accuracy ≥0.9, in order to obtain the circular consensus sequencing (CCS) reads. Subsequently, the lima (v 1.7.0) was employed to assign the CCS sequences to the corresponding samples based on their barcodes. CCS reads containing no primers and those reads beyond the length range (1,200–1,650 bp) were discarded through the recognition of forward and reverse primers and quality filtering using the cutadapt quality control process (v 2.7). The optimized sequence (tag) was obtained by screening and splicing. The optimized sequences are clustered and divided into OTUs (Operational Taxonomic Unit), and the OTU abundance information is normalized according to the normalized output data. Taxonomy information of bacterial OTUs was identified using the Ribosomal Database Project (RDP) classifier (http://rdp.cme.msu.edu/), and the UNITE (v 7.1) database were assigned to classify information of fungal OTUs (Abarenkov et al., 2010). The *α* diversity of bacterial and fungal communities was further analyzed. Picrust2 (v1.0) software was used to annotate the species by comparing the predicted characteristic sequence with the existing phylogenetic tree in the software. Integrated Microbial Genomes (IMG) database is used to output functional information, and then infer the functional gene composition in the sample, so as to analyze the functional differences between different samples or groups. First, it relies on an algorithm to insert marker sequences into existing phylogenetic trees with the help of short read layout tools. On this basis, the OTUs gene family was inferred. Then, it determines the gene family abundance of each sample. Finally, the sample path abundance is predicted by inferring the path abundance (Moussa, Maria & Géraldine, 2020). Ecological guilds of fungal communities were assigned using Fungi Functional Guild (FUNGuild v1.0) (http://fungu ild.org) (Nguyen et al., 2016). All sequences in the current study are stored in the sequence reading Archive (SRA) of NCBI database, with biological project ID: PRJNA735594 and accession number: SAMN19589874, SAMN19589875, SAMN19589876, SAMN19589877, SAMN19589878, SAMN19589879, SAMN19589880, SAMN19589881, SAMN19589882, SAMN19589883, SAMN19589884 and SAMN19589885.

### Statistical analysis

Soil physiochemical properties and plant diversity differences among different vegetation composition were tested by one-way analysis of variance (ANOVA) with Tukey multiple comparisons, and Pearson correlation analysis was performed to examine the *α* diversity relationships between microbial and plant. Permutational multivariate analysis of variance (PERMANOVA) analysis based on Jaccard distance matrices at microbial phylum and family levels was performed to compare difference of microbial taxonomic or functional composition among different vegetation composition. Redundancy analysis (RDA) was used to test the effects of measured environmental properties on microbial composition at both phylum and family levels (Edgar, 2013). All statistical analyses were performed using R programming language (v 4.0.3) with Vegan Packages.

## Results

### The composition and diversity of understory vegetation

The number of understory plant species under the forest with *C. japonica* is less than that without *C. japonica* (Table S2). We further compared the *α*-diversity indices and found similar evenness across the four relative abundance of *C. japonica* (Table 1). Notably, for the species richness and Shannon diversity, the highest values presented in moderate-proportion and the lowest in high proportion of *C. japonica*, whereas the Simpson’s dominance index was the greatest under high proportion of *C. japonica*. (*P* < 0.05, Table 1).

**Table 1:** The *α*-diversity of understory plant community under different proportion degree.

Degree of proportion	*E*_*H*_	*F*	*H*′	*D*
H	0.82 ± 0.03^a^	0.176 ± 0.00^b^	2.47 ± 0.04^b^	0.96 ± 0.003^a^
M	0.87 ± 0.01^a^	0.193 ± 0.00^a^	2.71 ± 0.05^a^	0.93 ± 0.009^b^
L	0.85 ± 0.00^a^	0.187 ± 0.00^ab^	2.68 ± 0.03^ab^	0.92 ± 0.003^b^
N	0.84 ± 0.00^a^	0.177 ± 0.00^b^	2.67 ± 0.05^ab^	0.92 ± 0.002^b^

**Notes.**

The data of different lowercase letters in the vertical row (mean and standard error) showed significant difference (*P* < 0.05).

Abbreviations *E*_*H*_ Pielou’s evenness index F Margalef richness index *H*′ Shannon diversity index D Simpson’s dominance index H High proportion (*C. japonica* trees account for 60% of the total plant individuals in the whole stand) M Moderate proportion (*C. japonica* account for 30% of the total plant individuals in the whole stand) L Low proportion (*C. japonica* account for 10% of the total plant individuals in the whole stand) N No proportion (no *C. japonica*)

### Changes of soil physiochemical properties

With the increase relative abundance of *C. japonica*, soil pH decreased significantly (*F* = 26.681, *df* = 3, DW (Durbin-Watson inspection) = 0.761, *P* < 0.01) (Table 2). The SOC content in forest with moderate *C. japonica* proportion was significantly higher than other proportion degrees (*F* = 57.732, *df* = 3, DW = 0.275, *P* < 0.01) and was as much as 32.5% higher than that without *C. japonica*. Soil TN, TK and SWC content increased significantly while AN, AP and AK showed a decrease trend alongside the increase of relative abundance of *C. japonica*. The content of soil TP changed little after *C. japonica* appearance (*F* = 6.191, *df* = 3, DW = 0.314, *P* = 0.981). These results suggested that most of the total soil nutrients increased while nutrient availability decreased with increase in the relative abundance of *C. japonica*.

**Table 2:** Differences in soil physicochemical properties of the top soil layer (0–20 cm) under different proportion degree.

Proportion degree	pH	SOC (g kg^−1^)	TN (g kg^−1^)	TP (g kg^−1^)	AN (mg kg^−1^)	AP (mg kg^−1^)	SWC (%)	TK (g kg^−1^)	AK (mg kg^−1^)
H	5.58 ± 0.06^c^	13.89 ± 0.25^c^	1.73 ± 0.01^a^	0.44 ± 0.02^a^	127.9 ± 2.13^c^	0.79 ± 0.04^a^	0.29 ± 0.02^a^	18.21 ± 0.20^a^	98.76 ± 0.21^b^
M	5.70 ± 0.05^b^	18.34 ± 0.41^a^	1.66 ± 0.04^b^	0.45 ± 0.02^a^	160.1 ± 3.52^a^	0.78 ± 0.07^a^	0.16 ± 0.01^b^	17.54 ± 0.11^b^	114.70 ± 0.16^b^
L	5.73 ± 0.02^b^	16.92 ± 0.43^b^	1.42 ± 0.02^c^	0.43 ± 0.01^a^	169.9 ± 4.73^a^	1.05 ± 0.32^a^	0.12 ± 0.01^c^	17.12 ± 0.23^b^	116.91 ± 0.20^b^
N	5.87 ± 0.01^a^	13.84 ± 0.18^c^	1.40 ± 0.01^c^	0.40 ± 0.01^a^	173.1 ± 2.01^a^	1.11 ± 0.02^a^	0.11 ± 0.01^c^	15.64 ± 0.18^c^	194.66 ± 0.28^a^
*P* value	0.001	0.004	0.001	0.981	0.001	0.56	0.036	0.025	0.552

**Notes.**

The data of different lowercase letters in the vertical row (mean and standard error) showed significant difference (*P* < 0.05).

Abbreviations H High proportion (*C. japonica* account for 60% of the total plant individuals in the whole stand) M Moderate proportion (*C. japonica* account for 30% of the total plant individuals in the whole stand) L Low proportion (*C. japonica* account for 10% of the total plant individuals in the whole stand) N No proportion (no *C. japonica*) SOC soil organic carbon TN total nitrogen TP total phosphorus TK total potassium AN available nitrogen AP available phosphorus AK available potassium SWC soil water content

### Composition of soil microbial community

**Figure 1:**
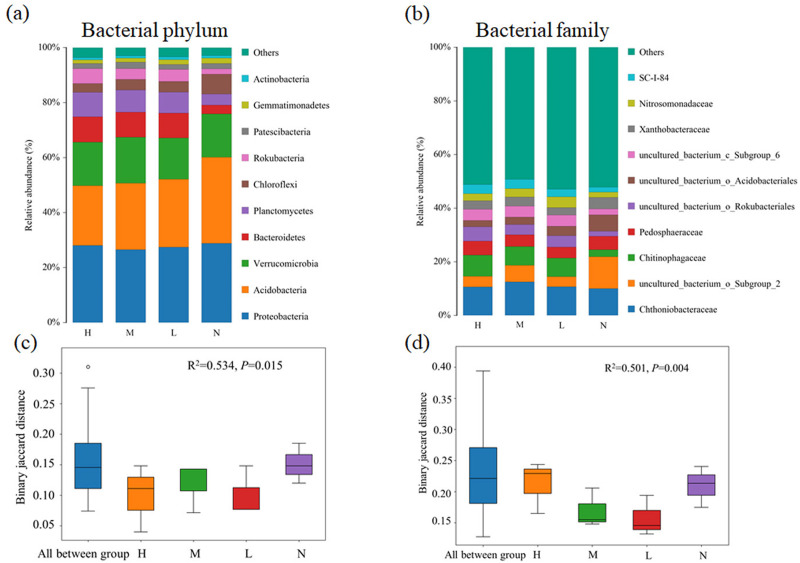
Changes of soil bacterial community composition across four degrees of *C. japonica* proportion. The community composition of soil bacteria at the level of phylum (A) and family (B) are presented and the significance of composition difference is illustrated. By PERMANOVA analysis based on phylum (C) and family (D) level. Abbreviations: H, High proportion (*C. japonica* account for 60% of the total plant individuals in the whole stand); M, Moderate proportion (*C. japonica* account for 30% of the total plant individuals in the whole stand); L, Low proportion (*C. japonica* account for 10% of the total plant individuals in the whole stand); N, No proportion (no *C. japonica*).

Proteobacteria (26.5%∼28.8%) was the most abundant bacterial phylum in almost all samples, followed by Acidobacteria (21.7%∼31.3%), Verrucomicrobia (15.0%∼16.7%) and Bacteroidetes (3.2%∼9.3%) (Fig. 1A). Compared with the broad-leaved forest with no *C. japonica*, the relative abundance of Acidobacteria in the broad-leaved forest with high proportion of *C. japonica* decreased by 9.6%, while the relative abundance of Bacteroidetes displayed an increase trend with the increase in *C. japonica* abundance. At family level, the relative abundance of uncultured_b acterium_o_Subgroup_2 decreased significantly after the emergence of *C. japonica* while that of the Chitinophagaceae increased markedly (Fig. 1B). The PERMANOVA analysis based on phylum (Fig. 1C) (*R*^2^ = 0.534, *P* = 0.015) and family (Fig. 1D) level (*R*^2^ = 0.501, *P* = 0.004) showed that with the increase of *C. japonica* proportion, the soil bacterial community changed significantly.

**Figure 2:**
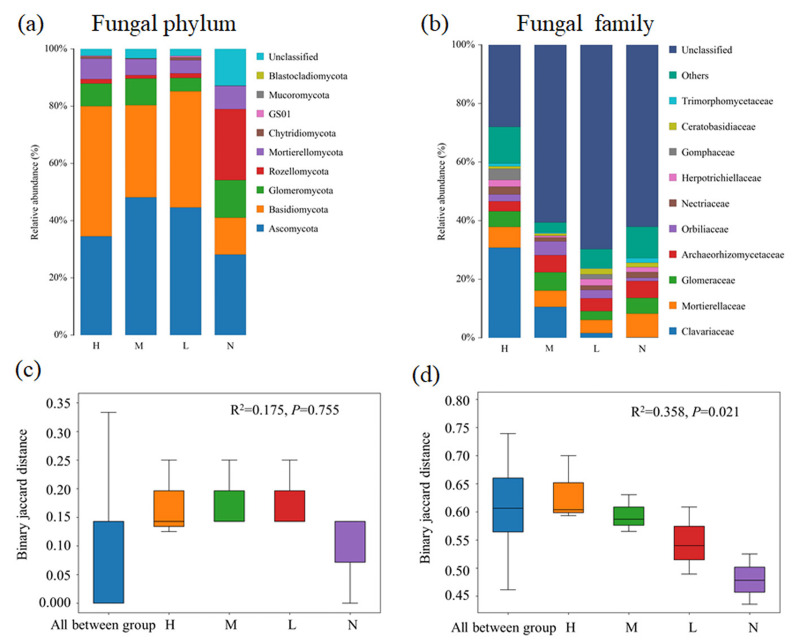
Changes of soil fungal community composition across four degrees of *C. japonica* proportion. The community abundance composition of fungi at the level of soil phylum (A) and family (B) are presented and the significance of composition difference is illustrated. By PERMANOVA analysis based on phylum (C) and family (D) level. Abbreviations: H, High proportion (*C. japonica* account for 60% of the total plant individuals in the whole stand); M, Moderate proportion (*C. japonica* account for 30% of the total plant individuals in the whole stand); L, Low proportion (*C. japonica* account for 10% of the total plant individuals in the whole stand); N, No proportion (no *C. japonica*).

Fungal composition at both phylum and family levels showed substantial changed with the increase of the proportion of *C. japonica*. In all soil samples, Basidiomycota and Ascomycota are dominant phylum. Obviously, Basidiomycota has an overwhelming advantage in forests with *C. japonica*, but Rozellomycota was the most abundant group in forest without *C. japonica* (Fig. 2A). At the family level, the relative abundance of Clavariaceae increases with the increase of the proportion of *C. japonica*. Compared with the broad-leaved forest soil with no *C. japonica*, the abundance of Clavariaceae in the broad-leaved forest soil with a high proportion of *C. japonica* increased by 30.63% (Fig. 2B). Overall, there were obvious differences of fungal taxonomic composition among forests with different proportions of *C. japonica* (*R*^2^ = 0.358, *P* = 0.021).

By comparing and annotating the KEGG database, six biological metabolic pathways were obtained by soil bacteria in broad-leaved forest (Fig. S3A). Among them, metabolism, genetic information processing and environmental information processing are important components of soil bacterial function of broad-leaved forest. Their relative abundances are 79.76% ± 0.81%, 7.46% ± 0.07% and 5.49% ± 0.07, respectively. The functional composition of bacterial community only showed slight shifts after the emergence of *C. japonica*. There were no significant differences of bacterial function composition among forests with different proportions of *C. japonica* (*R*^2^ = 0.358, *P* = 0.021) (Fig. S3B). The fungi functional group with different proportion degrees were mainly identified as pathotrophic (2.7%–10.7%), saprophytic (52.7%–58.9%) and symbiotic (30.3%–42.8%) (Fig. S4A). Saprophytic type was the most abundant one, and it more abundant in the forest containing *C. japonica*. The relative abundance of pathotrophic type decreased gradually with the increase of proportion degree, while the relative abundance of saprophytic type increased slightly after the emergence of *C. japonica*. There were no significant differences of fungal function composition among forests with different proportions of *C. japonica* (*R*^2^ = 0.282, *P* = 0.522) (Fig. S4B).

### Soil microbial *α* diversity

**Figure 3:**
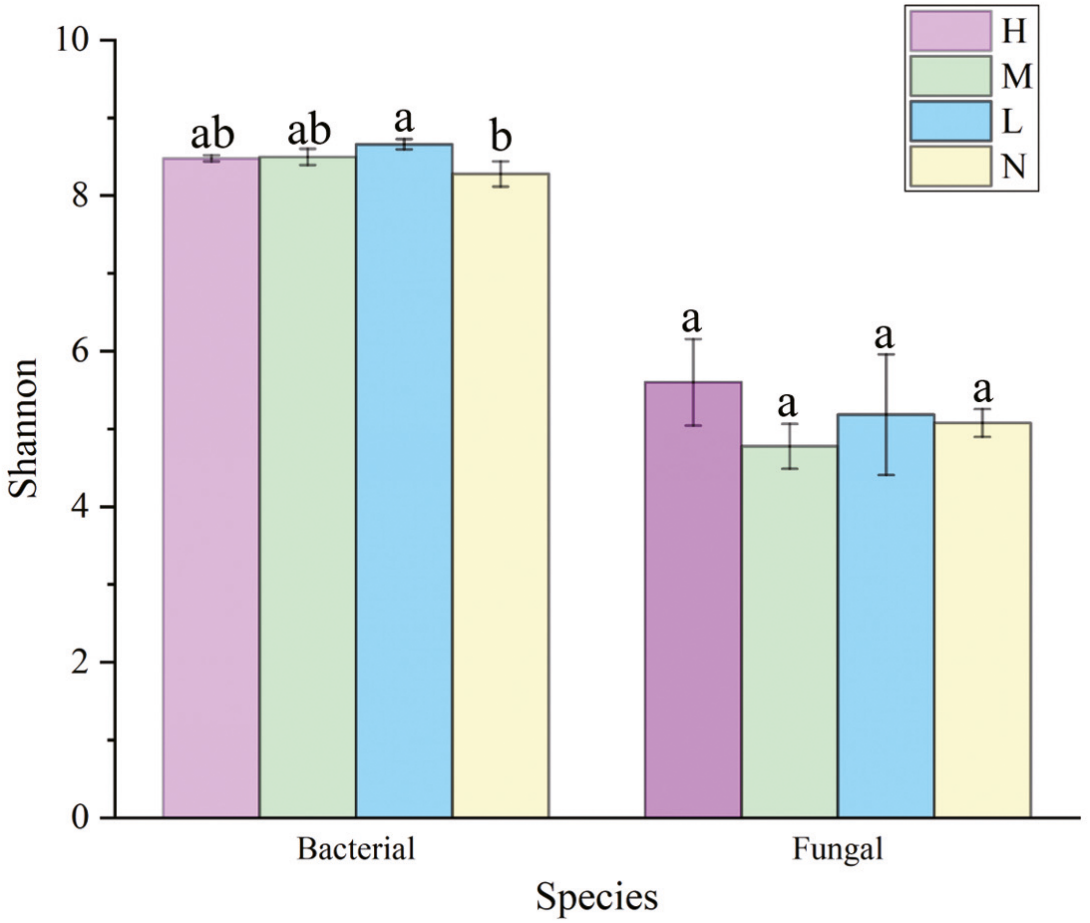
Variation of soil microbial *α*-diversity across four *C. japonica* proportion degrees. Abbreviations: H(B), Bacteria in high proportion; H(F), Fungal in High proportion (*C. japonica* account for 60% of the total plant individuals in the whole stand); M(B), Bacteria in Moderate proportion; M(F), Fungal in Moderate proportion (*C. japonica* account for 30% of the total plant individuals in the whole stand); L(B), Bacteria in Low proportion, L(F), Fungal in Low proportion (*C. japonica* account for 10% of the total plant individuals in the whole stand); N(B), Bacteria in No proportion; N(F), Fungal in No proportion (no *C. japonica*). Different lowercase letters of the same type (bacteria, fungi) show significant differences at the level of *P* ≤ 0.05.

The Shannon index of soil bacterial community was greater in forests harboring *C. japonica* compared to those without *C. japonica* (Fig. 3), and it was the greatest in the moderate proportion (S_M_ = 8.699 ± 0.87). There were no significant differences in soil fungi *α* diversity among different relative abundance of *C. japonica* (Fig. 3), although the Shannon diversity of fungal community under high *C. japonica* proportion (Shannon_H_ = 5.775 ± 0.29) was obviously higher than that under other proportion. The Simpson diversity of both soil bacterial and fungal communities had no significant differences among forests with various *C. japonica* abundance (Fig. S1).

Regression curves of plant diversity and microbial diversity was displayed in Fig. S2. A significant unimodal correlation between plant diversity and bacterial diversity (*P* = 0.036) was detected (Fig. S2A), and an extremely significant correlation between plant diversity and fungal diversity (*P* < 0.001) was observed (Fig. S2B).

### Correlations between soil environmental factors and microbial structures

**Figure 4:**
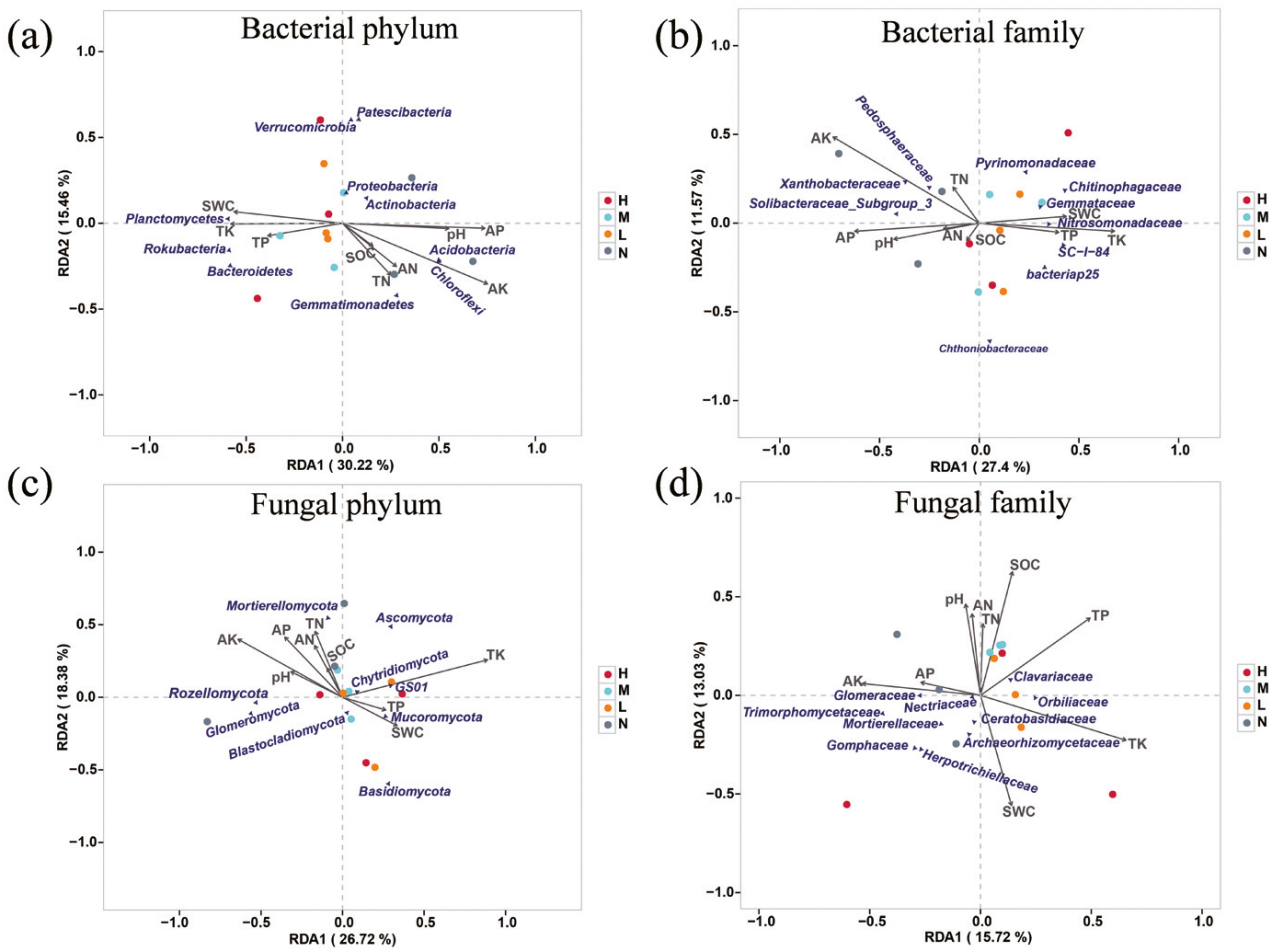
Redundancy analysis (RDA) examines the correlations between soil microbial structure and environmental factors. Associations of soil properties with bacterial structure at the level of phylum (A) and family (B) and fungal structure at the level of phylum (C) and family (D) were displayed. The soil properties that were significantly related to microbial structures are indicated by arrows, and microbial taxa and sampling sites were indicated by points.

Redundancy analysis was used to determine the impact of environmental factors on soil microbial community. The results showed that AK, AP, TK, SWC and pH had significant impacts on soil microbial composition (Fig. 4). A total of 45.68% of the variation of soil bacterial phylum structure could be explained by the first two components (Fig. 4A). Principal component one (27.4%) and two (11.57%) accounted for 38.97% of the total variation of soil bacterial family structure (Fig. 4B). AP, AK, TK were the main factors that affected soil bacterial community. Among them, AK and AP were positively correlated with family Pedosphaeraceae, Xanthobacteraceae and Soilbacteraceae_Subgroup_3, and TK was positively correlated with family Nitrosomonadaceae, Gemmataceae, Chitinophagaceae and SC-1-84. The first two principal components explained 45.1% variation of soil fungal community structure at phylum level, and AK, AP, TN, AN were positively correlated with Rozellomycota and Glomeromycota, while negatively related to Mucoromycota, GS01 and Basidiomycota (Fig. 4C). The contribution of the first two principal components to the variance of soil fungal family structure amounted to 28.75%. Specifically, AN, TN, pH and SOC were negatively correlated with Mortierellaceae, Herpotrichiellaceae and Gomphaceae but positively related to Clavariaceae. SWC was positively correlated with Archaeorhizomycetaceae and Ceratobasidiaceae (Fig. 4D).

### Correlation between vegetation structure and microbial structures

Redundancy analysis (RDA) was used to detect associations between microbial structure and vegetation features (Fig. S5). Results showed that bacterial composition at phyla and family levels and fungal composition at family level were positively correlated with plant Shannon diversity, Pielou, Richness, Vegetation coverage and Vegetation height (Figs. S5A, S5B and S5D). Fungi composition at phylum level was positively correlated with Shannon, Pielou, Richness and Vegetation height, and negatively correlated with Vegetation coverage (Fig. S5C). Plant species richness had the greatest impact on bacterial structure, and Vegetation coverage was the most important in affecting fungal structure.

## Discussion

### Changes of soil physicochemical properties and vegetation diversity after the change of relative abundance of *C. japonica*

Soil provides substrate and nutrient source for plant growth, and plants affect the element cycles in ecosystem through physical, chemical or biological processes. Studies have shown that plants can improve their competitiveness by changing soil nutrient contents and creating new habitats (Wang et al., 2012; Wang, Gong & Liu, 2015; Cao et al., 2019). Our results showed an increased SOC, TN, TP, TK and SWC while decreased pH with increase of *C. japonica* abundance in broad-leaved forest in karst area. However, it is generally believed that the sudden emergence of new species will consume a lot of soil nutrients, leading to the depletion of soil nutrients (Zhu & Wu, 2012). Our results also supported this speculation, as indicated by significant decreases in available nutrients content, including AN, AP and AK, Further proof that the rapid increase of the relative abundance of *C. japonica* may depend on the rapid consumption of soil available nutrients, rather than a native forest soil total nutrients. Another reason for the declined nutrients availability might be that the increase of relative abundance of *C. japonica* induced soil microbial shifts towards a community that can stimulate soil nutrient immobilizations (Figs. 1 and 2). In addition, after the emergence of *C. japonica*, the number of undergrowth plants decreased, and the soil erosion increased. Such changes in soil properties probably cause losses in available nutrients that are easily to be leached. Soil TP content is greatly affected by soil parent material and soil formation processes, the content of exchangeable cations in the litters of *C. japonica* might be lower than that of the native species, thus affecting the mineral weathering of parent material (Lin, Peng & Hong, 2017). The decrease of soil pH may be due to the increase of soil acidity caused by acidic substances in the litter and root exudates of *C. japonica*. The increase of soil water content was mainly resulted from the improvement of soil structure and the enhancement of soil water storage capacity with the increase of SOC content. In addition, the emergence of *C. japonica* can reduce the canopy width, light transmittance and water evaporation. Moreover, *C. japonica* has the characteristics of soil and water conservation, which could contribute to soil water content increases.

Species diversity is commonly used to reflect the dynamic changes of communities in the ecological processes (Hao et al., 2014). Our study showed that Shannon diversity of understory species were the lowest under a higher *C. japonica* abundance. This might be due to the homogenization of understory environments due to increase of forest canopy density and decrease of light resources (Maureen et al., 2007; Yu & Sun, 2013; Huang et al., 2018). Meanwhile, due to the rapid growth of *C. japonica*, the living space of understory plants was restricted, which may contribute to the losses of original species. In addition, the herb layer showed greater changes in species composition than shrub layer. This result might be owing to the fact that the life history of herb layer plants is shorter than that of shrub layer plants, and they have stronger adaptability and wider niche, making the species composition of community unstable and variable (David, 2002).

### Changes in soil microbial community composition and diversity after the change of relative abundance of *C. japonica*

As an important part of forest ecosystem, soil microorganism can play a leading role in nutrient and energy flow of soil ecosystem (Richardson, 2001). When the external environment changes, soil microbial species diversity (Lesaulnier et al., 2008), community structure (Li et al., 2010), and functional composition (Zheng et al., 2012) will change accordingly. However, here we only found a significant shifts in soil bacterial and fungal taxonomic structure but little in their functional structure after emergence of *C. japonica* in a broad-leaved forest in the karst area. For example, the relative abundance of Proteobacteria increased after the emergence of *C. japonica*. This may be due to the increased SOC content, as Proteobacteria is generally considered to be an R-strategy species with a preferential advantage in habitats rich in organic matter (Rodrigues, Pellizari & Mueller, 2013; He et al., 2016). After emergence of *C. japonica*, the abundance of Acidobacteria decreased with decline in soil available nutrients, which was conflict with the recognition that Acidobacteria were oligotrophic microorganisms preferring nutrient poor environments (Magill & Aber, 2000; Huang, Tian & Jiang, 2009; Dai, Yan & Xie, 2017). However, previous studies also demonstrated a negative correlation of Acidobacteria with SWC and TP content (Cai et al., 2018). This partly explained the decrease of Acidobacteria in our results, as the SWC and TP showed an increased trend with the increase of the relative abundance of *C. japonica* (Table 2). Therefore, Acidobacteria might be more sensitive to changes in SWC and TP than the changes in other soil physicochemical properties. Moreover, the growth rate of Acidobacteria is relatively slow, when soil nutrient content changes, their fast-growing counterparts, such as Proteobacteria, will overwhelm oligotrophic Acidobacteria. Regarding to fungal community, the increase of relative abundance of *C. japonica* significantly stimulated growth of Basidiomycetes and Ascomycetes, but restricted growth of Rozellomycota. Basidiomycetes and Ascomycetes have a strong ability to decompose lignocellulose in plant residues (Beimforde et al., 2014; Riley et al., 2014), and they may be capable of inhibiting growth of other fungi *via* competing for carbon resources. In addition, the Ascomycetes are widely distributed in acid environments (Du et al., 2020), thus the decreased soil pH after the emergence of *C. japonica* may partly explain the increase of Ascomycetes abundance. We did not detect any significant differences in functional composition across various *C. japonica* abundance (Figs. S3 and S4). This unexpected finding was possibly attributed to the following reasons: (1) the emergence of *C. japonica* may introduce new soil microbial species closely related to roots and promote the expansion of *C. japonica*, thus this study focusing on bulk soil microorganisms may underestimate the changes in rhizosphere microorganisms with high functional diversity; (2) the reassembly of soil microbial communities attributed to changes in plant composition may not necessarily cause shifts in microbial functional composition due to microbial functional redundancy; (3) it is also important that the microbial functional composition is identified at the DNA level that cannot discern the active and inactive microbial groups, which may overestimate the functional expression. Thus, the RNA sequencing will be required in further studies to validate our results. In the future, we will further carry out comprehensive experiments to draw mechanisms underlying the different responses between taxonomic and functional structure of soil microbial community, especially the microbial functional groups that have not yet been defined and explained but may play a role after the change of vegetation composition.

### Relationships between microbial community and soil physicochemical properties

Soil nutrients content affect microbial community by directly influencing their foods and energy supply, and indirectly alter microbial metabolic activity by affecting nutrient cycling and plant growth (Wang et al., 2016; Zi et al., 2017; Niu et al., 2017). Here we found the relative abundance of Acidobacteria decreased while the Proteobacteria increased after emergence of *C. japonica*. Proteobacteria and Acidobacteria are widely distributed in soil systems around the world and are recognized as the most abundant dominant bacterial groups. Due to their different lifestyles, they are often used as indicators of soil nutrient status (Liu, Dong & Jiao, 2016). Acidobacteria grow in oligotrophic mode while Proteobacteria prefer soil environment rich in nutrients, and the latter can grow and metabolize with nutrients obtained from organic matter decomposition (Dong, Gao & Zhou, 2021). Unexpectedly, the relative abundance of Acidobacteria decreased after the emergence of *C. japonica* with declined AN, AK and AP. Perhaps Acidobacteria are more sensitive to the changes in soil total nutrients content instead of availably nutrients content. For instance, Wei et al. (2018) found that the relative abundance of Acidobacteria in rhizosphere soil strongly depended on total nitrogen content. The higher relative abundance of Proteobacteria in soil samples after the emergence of *C. japonica* mainly because of their strong adaptability to increased SOC quantity and quality caused by elevated understory plant diversity (Fan, An & Liang, 2021). In addition, the relative abundance of Chloroflexi decreased after the emergence of *C. japonica*, which was mainly attributed to the decreased nutrients content, as indicated by positive correlations with AK, AP, AN and TN. Previous research (Li, Hou & Wang, 2018) showed that Chloroflexi obtained energy through photosynthesis, therefore a continuous supply of soil nutrients, such as N and P was necessary for the synthetize of enzymes involved in photosynthesis. Regarding to the bacterial families, we observed that the enrichment of Chitinophagaceae and SC-I -84 after *C. japonica* emergence were positively correlated with TK and negatively correlated with AK and AP, which was consistent with previous study suggesting that the increase of these two families may be the key factor leading to the significant increase of soil TK (Gao et al., 2021).

Some researchers pointed out that SOC and TP had the greatest impact on soil fungal community structure (Chen et al., 2018; Cao et al., 2016; Kumar et al., 2019). Similarly, here the most abundant fungi (Ascomycota and Basidiomycetes) were substantially increased by *C. japonica* emergence and positively correlated with TK, TN, TP and SOC. Clavariaceae with quite higher abundance in the soil under *C. japonica* is positively correlated with SOC, AN and pH. Most of Ascomycetes and Basidiomycetes are saprophytic or parasitic fungi, which contribute significantly to the degradation of lignin, keratin and other recalcitrant organic matter in soil (Huang et al., 2008; Beimforde et al., 2014; Jiang et al., 2021). Clavariaceae, also belongs to saprophytic fungal, plays an important role in the decomposition of pectin and cellulose in forest litter (Li, Han & Wu, 2021). Undoubtedly, the saprophytic function expression of these fungi will enable them to survive soil conditions deplete in available nutrients while rich in total organic nutrients as the case of degraded broad-leaved forests in karst areas caused by the continuous expansion of *C. japonica*. Based on these results, we speculate that the fast spread of *C. japonica* in broad-leaved forests of the karst area will not change the potential ecological functions of the whole soil microbial community in broad-leaved forest, but may change the specific groups with certain ecological functions (Ma et al., 2021).

## Conclusion

In this study, we characterized the changes of soil bacterial and fungal community structure, soil physicochemical properties and understory plant features after the emergence of *C. japonica* in a broad-leaved forest in karst area. Meanwhile, relationships among microbial structure, soil properties and plant diversity were examined and compared under different relative abundance of *C. japonica* to try to explore the potential ecological implications derived from soil microbial changes. We found significant decreases in soil available nutrients content in parallel with shifts of bacterial taxonomic composition alongside the increase of *C. japonica* abundance. Changes in SOC, SWC and pH, were mainly related to shifts in fungal taxonomic composition. Moreover, Acidobacteria, Chloroflexi, Ascomycota and Basidiomycota are significantly correlated with soil property changes caused by emergence of *C. japonica*. Thus, soil bacterial and fungal communities both play important roles in shaping soil physicochemical environments. The rehabilitation of both bacterial and fungal community structure, in particular the taxonomic structure, should be considered during the effective restoration of soil ecological processes of the infertile karst forests caused by fast spread of *C. japonica*. These results provide novel insights into microbial regulating mechanisms of karst forest degradation under expansion of *C. japonica*.

## Supplemental Information

10.7717/peerj.12739/supp-1 Supplemental Information 1 Raw data for tree community composition and soil properties Raw data from the study presented as tree community composition matrix and soil physiochemical values

10.7717/peerj.12739/supp-2 Supplemental Information 2 Bacterial sequences

10.7717/peerj.12739/supp-3 Supplemental Information 3 Fungal sequences

10.7717/peerj.12739/supp-4 Supplemental Information 4 Supplementary figures and tables

## Additional Information and Declarations

### DNA Deposition

The following information was supplied regarding the deposition of DNA sequences: The soil microbial sequences are available at GenBank: SAMN19589874, SAMN19589875, SAMN19589876, SAMN19589877, SAMN19589878, SAMN19589879, SAMN19589880, SAMN19589881, SAMN19589882, SAMN19589883, SAMN19589884 and SAMN19589885.

### Data Availability

The following information was supplied regarding data availability: The data is available at NCBI: PRJNA735594.
