# Supplementary material for: Response of soil microbial community to plant composition changes in broad-leaved forests of the karst area in Mid-Subtropical China
Source: PeerJ. 2022 Mar 7;10:e12739. doi: 10.7717/peerj.12739 (PMC8908884; doi:10.7717/peerj.12739)
Supplement: Supplemental Information 4 [file peerj-10-12739-s004.docx]

**Supplementary materials for**

Response of soil microbial community to plant composition changes in broad-leaved forests of the karst area in Mid-Subtropical China

Liling Liu^1#^, Ninghua Zhu^1#^, Guangyi Zhou^2^, Peng Dang^1^, Xiaowei Yang^1^, Liqiong Qiu^1^, Muyi Huang^1^, Yingyun Gong^1^, Suya Zhao^1^, Jie Chen^2^*

^1^Faculty of forestry; Central South University of Forestry and Technology, Changsha, 410004, PR China

^2^Research Institute of Tropical Forestry, Chinese Academy of Forestry, Longdong, Guangzhou, 510520, PR China

# The first two authors contribute equally to the study

*Correspondence:

Dr. Jie Chen

Tel: + 86 20 8703 2619

Fax: +86 20 8703 1622

E-mail: [chenjiecaf@hotmail.com](mailto:chenjiecaf@hotmail.com)

**Table S1** Overview of the study area.

| Degree of proportion | Attitude (m) | Slope (°) | Average tree height (m) | | Mean DBH (cm) | | Density（plant/hm^2^） | |
| --- | --- | --- | --- | --- | --- | --- | --- | --- |
|  |  |  | *C. japonica* | broad-leaved forest | *C. japonica* | broad-leaved forest | *C. japonica* | broad-leaved forest |
| H | 651 | 18 | 10.2 | 16.7 | 9.3 | 15.8 | 1487 | 960 |
| M | 635 | 21 | 7.5 | 17.2 | 10.2 | 18.7 | 538 | 1100 |
| L | 620 | 18 | 5.6 | 18.0 | 7.5 | 19.9 | 156 | 1120 |
| N | 612 | 19 | 0 | 18.2 | 0 | 21.6 | 0 | 1250 |

Abbreviations: H: High proportion (*C. japonica* account for 60% of the total plant individuals in the whole stand); M: Moderate proportion (*C. japonica* account for 30% of the total plant individuals in the whole stand); L: Low proportion (*C. japonica* account for 10% of the total plant individuals in the whole stand); N: No proportion (no *C. japonica*).

**Table S2** The taxonomic information of understory vegetation under different proportion degree.

| Degree of proportion | Structure | Family | Genus | Species |
| --- | --- | --- | --- | --- |
| H | Shrub layer | 15 | 13 | 14 |
|  | Herb layer | 14 | 19 | 20 |
| M | Shrub layer | 10 | 13 | 16 |
|  | Herb layer | 17 | 22 | 22 |
| L | Shrub layer | 15 | 18 | 20 |
|  | Herb layer | 16 | 26 | 27 |
| N | Shrub layer | 16 | 22 | 27 |
|  | Herb layer | 13 | 25 | 26 |

Abbreviations: H: High proportion (*C. japonica* account for 60% of the total plant individuals in the whole stand); M: Moderate proportion (*C. japonica* account for 30% of the total plant individuals in the whole stand); L: Low proportion (*C. japonica* account for 10% of the total plant individuals in the whole stand); N: No proportion (no *C. japonica*).


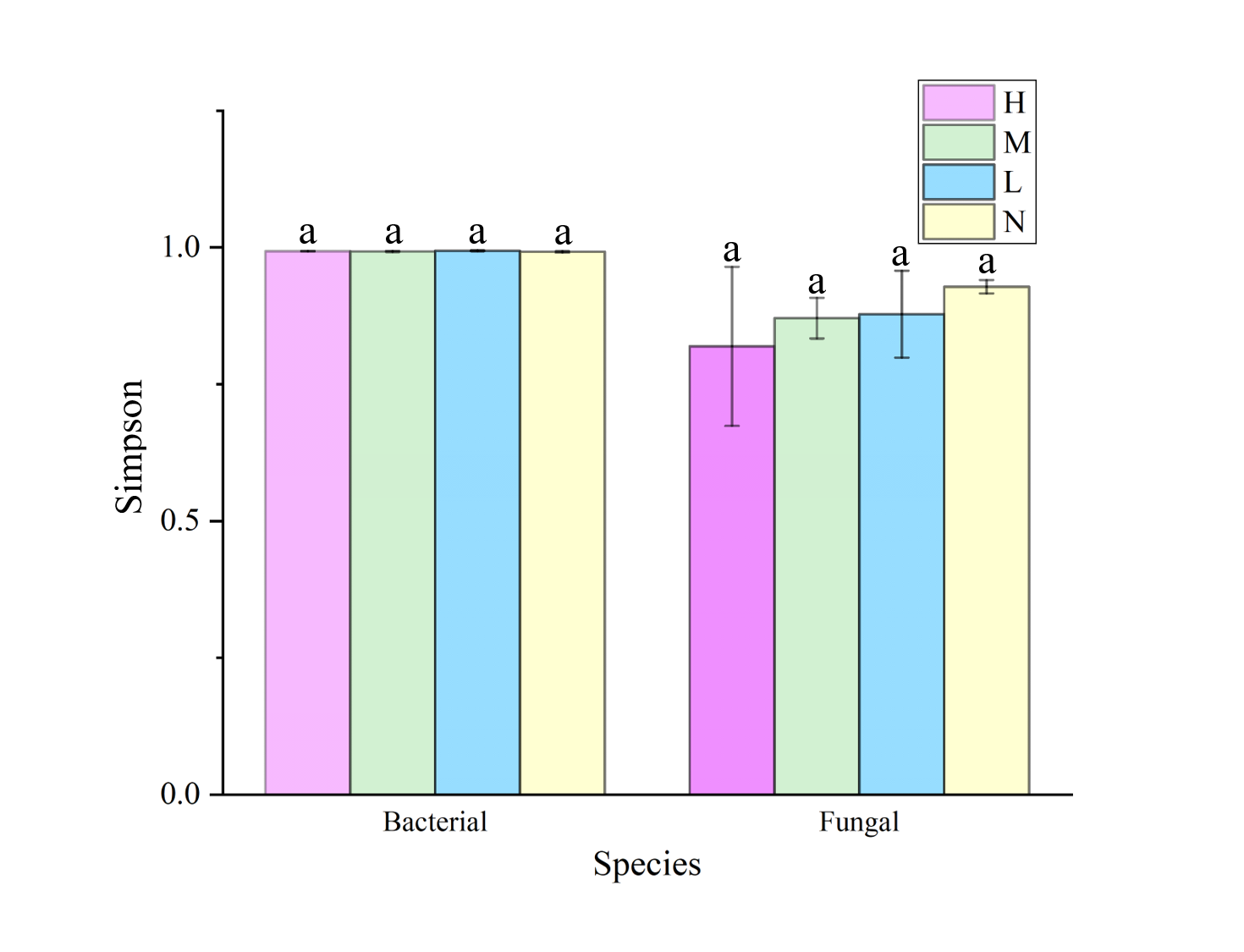


**Figure S1** Variation of soil microbial Simpson index across four *C. japonica* proportion degrees. Abbreviations: H: High proportion (*C. japonica* account for 60% of the total plant individuals in the whole stand); M: Moderate proportion (*C. japonica* account for 30% of the total plant individuals in the whole stand); L: Low proportion (*C. japonica* account for 10% of the total plant individuals in the whole stand); N: No proportion (no *C. japonica*). Different lowercase letters of the same type (bacterial, fungal) show significant differences at the level of *P*=0.05.


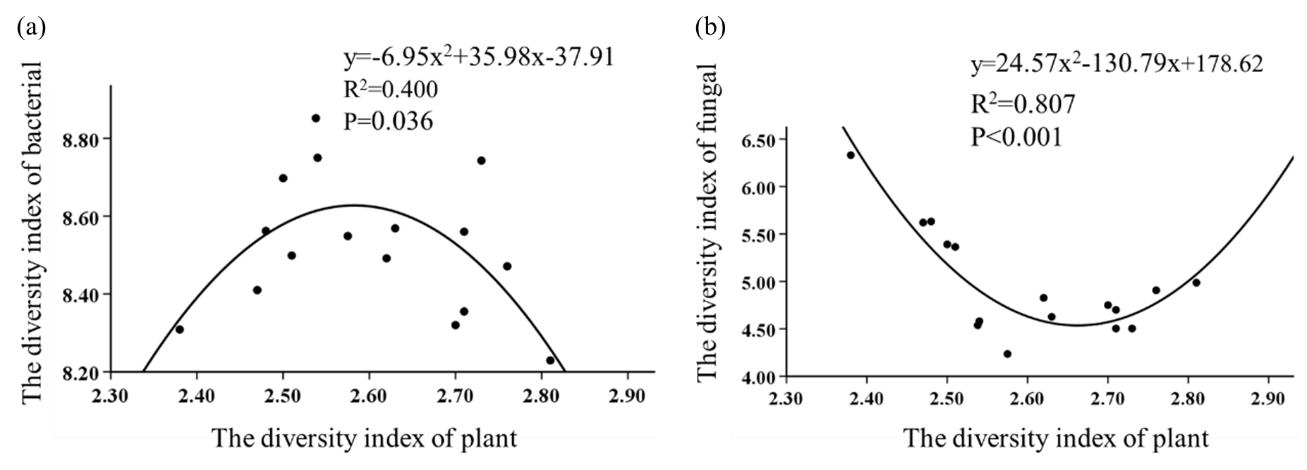


**Figure S2** The correlation curves between microbial and plant Shannon diversity. Regression curves of plant diversity and microbial diversity was displayed in Figure S2. A significant unimodal correlation between plant diversity and bacterial diversity (*P* = 0.036) was detected (Fig.S2a), as indicated by a first increase and then decrease of bacterial diversity with the increase of plant diversity. An extremely significant correlation between plant diversity and fungal diversity (*P* < 0.001) was observed (Fig.S2 b), with a first decrease and then increase of fungal diversity alongside the increase of plant diversity.

**Figure S3** Changes of soil bacterial functional composition across different proportion degree of *C. japonica*. Community composition difference was tested by PERMANOVA analysis based on function level (Fig. S3b). By comparing KEGG database, picrust2 gene was used to predict 7 kinds of metabolic pathways: Metabolism, Genetic information processing, Environmental information processing, Cellular processes, Organismal systems, Human diseases and Organismal Systems (Fig. S3a). The relative abundance of these functional genes kept stable after the emergence of *C. japonica*, regardless of proportion degree. Abbreviations: H: High proportion (*C. japonica* account for 60% of the total plant individuals in the whole stand); M: Moderate proportion (*C. japonica* account for 30% of the total plant individuals in the whole stand); L: Low proportion (*C. japonica* account for 10% of the total plant individuals in the whole stand); N: No proportion (no *C. japonica*).


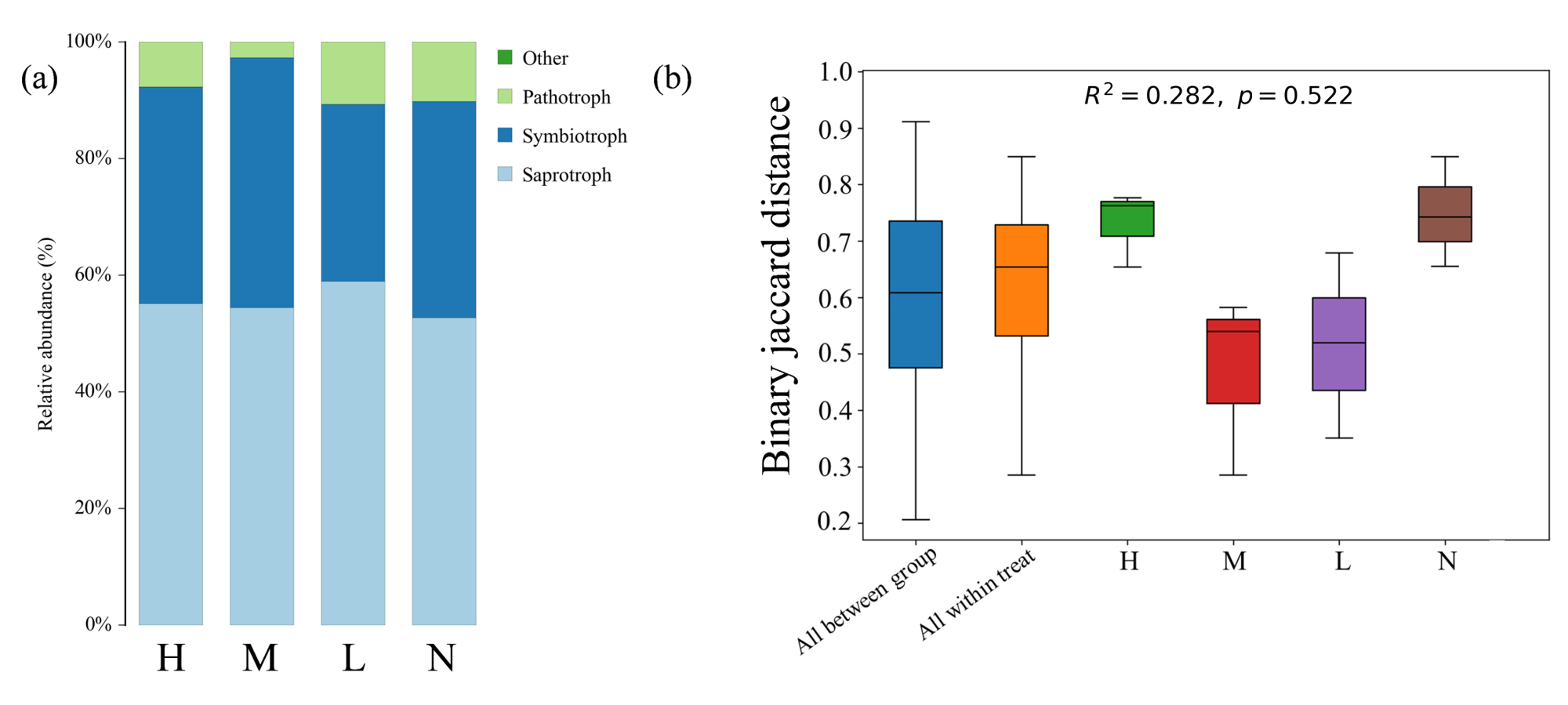


**Figure S4** Changes of soil fungal functional composition across different proportion degree of *C. japonica*. Community composition difference was tested by PERMANOVA analysis based on function level (Fig. S4b). Abbreviations: H: High proportion (*C. japonica* account for 60% of the total plant individuals in the whole stand); M: Moderate proportion (*C. japonica* account for 30% of the total plant individuals in the whole stand); L: Low proportion (*C. japonica* account for 10% of the total plant individuals in the whole stand); N: No proportion (no *C. japonica*).

**Figure S5** Redundancy analysis (RDA) examines the correlations between soil microbial structure and vegetation structure. Associations of vegetation structure with bacterial structure at the level of phylum (a) and family (b) and fungal structure at the level of phylum (c) and family (d) were displayed. The vegetation structure that were significantly related to microbial structures are indicated by gray arrows, and microbial taxa and sampling sites were indicated by points.
